# Sample size estimation for AQP4-IgG seropositive optic neuritis: Retinal damage detection by optical coherence tomography

**DOI:** 10.1515/biol-2022-0866

**Published:** 2024-04-16

**Authors:** Shuwen Lu, Chao Ma, Yi Du

**Affiliations:** Department of Ophthalmology, The First Affiliated Hospital of Henan University of Traditional Chinese Medicine, No. 19 Renmin Road, Zhengzhou 450099, China; Department of Ophthalmology, The First Affiliated Hospital of Zhengzhou University, No. 1 Jianshe East Road, Zhengzhou 450052, China; Department of Ophthalmology, The First Affiliated Hospital of Guangxi Medical University, No. 6 Shuangyong Road, Nanning 530021, China

**Keywords:** sample size, optical coherence tomography, aquaporin-4, optic neuritis

## Abstract

We recruited four aquaporin-4 seropositive optic neuritis patients (five eyes) who received glucocorticoid treatment and underwent optical coherence tomography examination. Baseline medians of the macular ganglion cell layer plus inner plexiform layer (mGCIPL) thickness and volume for the eye of interest were 79.67 µm (73.664 ± 18.497 µm) and 0.58 mm^3^ (0.534 ± 0.134 mm^3^), respectively. At 2 months, the medians of the mGCIPL thickness and volume were 60.00 µm (51.576 ± 12.611 µm) and 0.44 mm^3^ (0.376 ± 0.091 mm^3^), respectively. At 6 months, the medians of the mGCIPL thickness and volume were 59.55 µm (46.288 ± 11.876 µm) and 0.44 mm^3^ (0.336 ± 0.084 mm^3^), respectively. Sample size estimate was achieved using two methods based on the mGCIPL thickness and volume data, with five effect sizes considered. The estimate based on the mGCIPL volume showed that 206 patients were needed at the 6-month follow-up; the power was 80% and effect size was 20%. In conclusion, this study detected retinal damage in aquaporin-4 seropositive optic neuritis patients by optical coherence tomography, and estimated the sample size for two-sample parallel designed clinical trials using two methods.

## 1 Introduction

Optic neuritis (ON) is an autoimmune inflammatory demyelinating disease of the optic nerve that primarily affects young people aged 18–45 years, frequently resulting in permanent visual dysfunction [1]. Aquaporin-4 (AQP4) immunoglobulin G (IgG) seropositive ON patients suffer more severe vision loss and exhibit poor vision recovery, compared to AQP4 IgG seronegative ON patients [2]. Retinal ganglion cell (RGC) loss as a main feature of the irreversible retinal injury has been shown in the eyes of ON patients [3]. More than 50% of the macular cells are RGCs, and thinning of the macular layers, which are composed of the retinal nerve fiber layer (RNFL) and the ganglion cell layer plus inner plexiform layer (GCIPL), was observed in ON and ischemic optic neuropathy [4,5,6]. Optical coherence tomography (OCT) can be applied to non-invasively measure the RNFL and GCIPL thicknesses in neurological diseases [7]. Considering that the distribution of the RNFL exhibits distinct differences between individuals, the distribution of the GCIPL is relatively stable [8], thus, measuring the macular GCIPL (mGCIPL) in ON patients is more meaningful.

The dominant view is that glucocorticoid (GC) treatment of ON does not improve the patients’ final visual acuity, and developing optic nerve protection drugs is one of the most important objectives of current research. Performing randomized controlled trials is one of the most important methods to evaluate the efficacy and safety of new interventions. To ensure reliable results, avoid abusing resources and minimize damage, sample sizes of randomized controlled trials require estimation. However, previous studies estimating the sample size for ON did not distinguish between AQP4-IgG seropositive and seronegative patients [9,10]. Therefore, in this study, we aim to estimate the sample size for two-sample parallel designed clinical trials based on the mGCIPL volume and thickness in AQP4-IgG seropositive ON patients, which will provide a reference for the future study of neuroprotective or myelin regenerative agents.

## 2 Participants and methods

### 2.1 Participants

**Figure 1:**
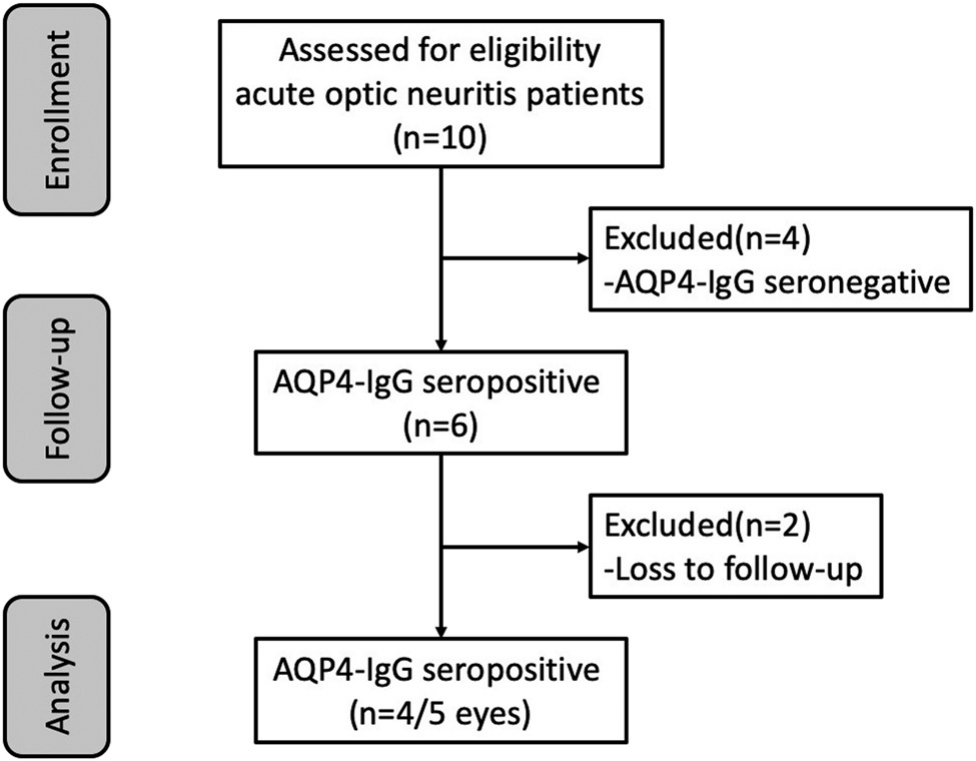
Flow diagram of participants.


Four eligible patients (one male and three females, median age 42 years, range 22–51 years, Table 1) were recruited from the First Affiliated Hospital of Guangxi Medical University from January 2017 to January 2019. The median time of the first OCT examination after the onset of symptoms was 12 days, ranging from 10 to 16 days. The patient inclusion criteria were: (1) First episode of acute ON. (2) AQP4-IgG seropositive. (3) Chinese patients aged 18–60 years without gender limitation. (4) Best-corrected visual acuity of the eye of interest ≤0.8. Exclusion criteria included any of the following: (1) Patients with pre-existing multiple sclerosis (MS) or neuromyelitis optica. (2) Refractive media opacity, hyperopia >5 diopters, myopia <−5 diopters or astigmatism >3 diopters. (3) Active tuberculosis, hepatitis, renal insufficiency, uncontrolled hypertension, diabetes mellitus, HIV or syphilis infection or any other conditions potentially interfering with the treatment trial. (4) Other autoimmune diseases (systemic lupus erythematosus, rheumatoid arthritis and other diseases). (5) Existing other retinal or optic nerve diseases. The flow diagram of participants is shown in Figure 1.

**Table 1:** Characteristics of eyes included

Case	laterality	Baseline		2 months		6 months	
		Mean of mGCIPL volume (mm^3^)	Mean of mGCIPL thickness (μm)	Mean of mGCIPL volume (mm^3^)	Mean of mGCIPL thickness (μm)	Mean of mGCIPL volume (mm^3^)	Mean of mGCIPL thickness (μm)
1	OD	0.62	87.77	0.46	64.11	0.37	53.44
2	OD	0.30	41.66	0.25	33.77	0.25	32.55
3	OS	0.58	79.67	0.44	60.00	0.44	59.55
4	OD	0.55	75.22	0.31	43.44	0.25	35.01
5	OS	0.62	84.00	0.42	56.56	0.37	50.89

OD represents for right eye and OS represents for left eye.

**Informed consent:** Informed consent has been obtained from all individuals included in this study.
**Ethical approval:** The research related to human use has been complied with all the relevant national regulations, institutional policies and in accordance with the tenets of the Helsinki Declaration, and has been approved by the ethical review committee of First Affiliated Hospital of Guangxi Medical University (No. 2017(KY-E-068)).

### 2.2 AQP4-IgG test and ophthalmologic examinations

The serum was tested for AQP4-IgG by a cell-based assay. Every patient underwent ophthalmologic examinations and were given methylprednisolone intravenously for 3–5 days, the dosage subsequently being gradually reduced. The eye examination at onset and at 2- and 6-month follow-up included the best-corrected visual acuity, pattern visual evoked potentials and visual field examination.

### 2.3 OCT examinations and analyses

Retinal images were obtained on the day of hospitalization by Spectralis OCT (Heidelberg Engineering, Heidelberg, Germany), which provides 40,000 A-scans per second with a 5.7 µm digital axial resolution. The following retinal layers were produced: (1) RNFL; (2) mGCIPL; (3) inner nuclear layer and outer plexiform layer; and (4) outer nuclear layer (including inner and outer photoreceptor segments). Briefly, macular data scans were obtained with Macular Cube 512 × 128 protocols. The software (Spectralis OCT) automatically generates thickness maps corresponding to the foveal area and the paracentral mGCIPL. The software (Spectralis OCT) also identifies the inner limiting membrane and the inner boundary of the retinal pigment epithelium within the 6 mm × 6 mm × 1.9 mm volume cube scan to determine the mGCIPL volume. The images were obtained of the patients with dilated pupils (Compound Tropicamide Eye Drops: 0.5% tropicamide mixed with 0.5% deoxyepinephrine hydrochloride in a 1:1 ratio) by the same trained technician. All images were reviewed by a trained physician to ensure appropriate scan quality and correct segmentation. If the segmentation was incorrect, a manual correction was performed.

### 2.4 Statistical analyses and sample size estimation

All statistical analyses and sample size estimation were completed using the R program (R for Mac OS X GUI 3.4.3). The median and mean values and standard deviation (SD) were obtained to describe the data distribution. Differences were considered to be statistically significant for *P*-values < 0.05. The sample size estimate was achieved using the following equations:

κ=nGCs ntest drug,

$$n_{\mathrm{test drug}}=\left(1+\frac{1}{\kappa }\right){\left(\sigma \frac{Z_{1-\frac{\alpha }{2}}+Z_{1-\beta }}{\mu_{\mathrm{GCs}}-\mu_{\mathrm{test drug}}}\right)}^{2},$$

$$1-\beta =\phi (Z-Z_{1-\frac{\alpha }{2}})+\phi \left(\;,-Z-Z_{1-\frac{\alpha }{2}}\right),$$

$$Z=\frac{\mu_{\mathrm{GCs}}-\mu_{\mathrm{test drug}}}{\sigma \sqrt{1/n_{\mathrm{GCs}}+1/n_{\mathrm{test drug}}}},$$

where *κ* is the matching ratio; *σ* is the SD; *ϕ* is the standard normal distribution function; *α* is the Type I error (0.05); *β* is the Type II error, and 1 − *β* is the power (80, 90, and 95%).

Each patient underwent OCT examination on certain dates. The sample size was estimated using the following two methods:

*Method A*: Comparison of the follow-up of the affected eye mGCIPL thickness and volume. The mean and SD values of the mGCIPL volume and thickness at the follow-up of the affected eye were required. Assuming that the protective effects of the test drug on the mGCIPL thickness and volume were 20, 30, 40, 50 and 60% at 2 and 6 months, the mean values for the test drug at 2 and 6 months were calculated according to the different effect sizes of therapeutic effects. Subsequently, the sample size was estimated.

*Method B*: Comparison of the difference between the follow-up and baseline of the affected eye mGCIPL volume and thickness. The SD values of this difference were required. The difference was obtained from the baseline value minus the follow-up value. The other parts were consistent with *Method A*.

## 3 Results

### 3.1 OCT and ophthalmic examinations

**Figure 2:**
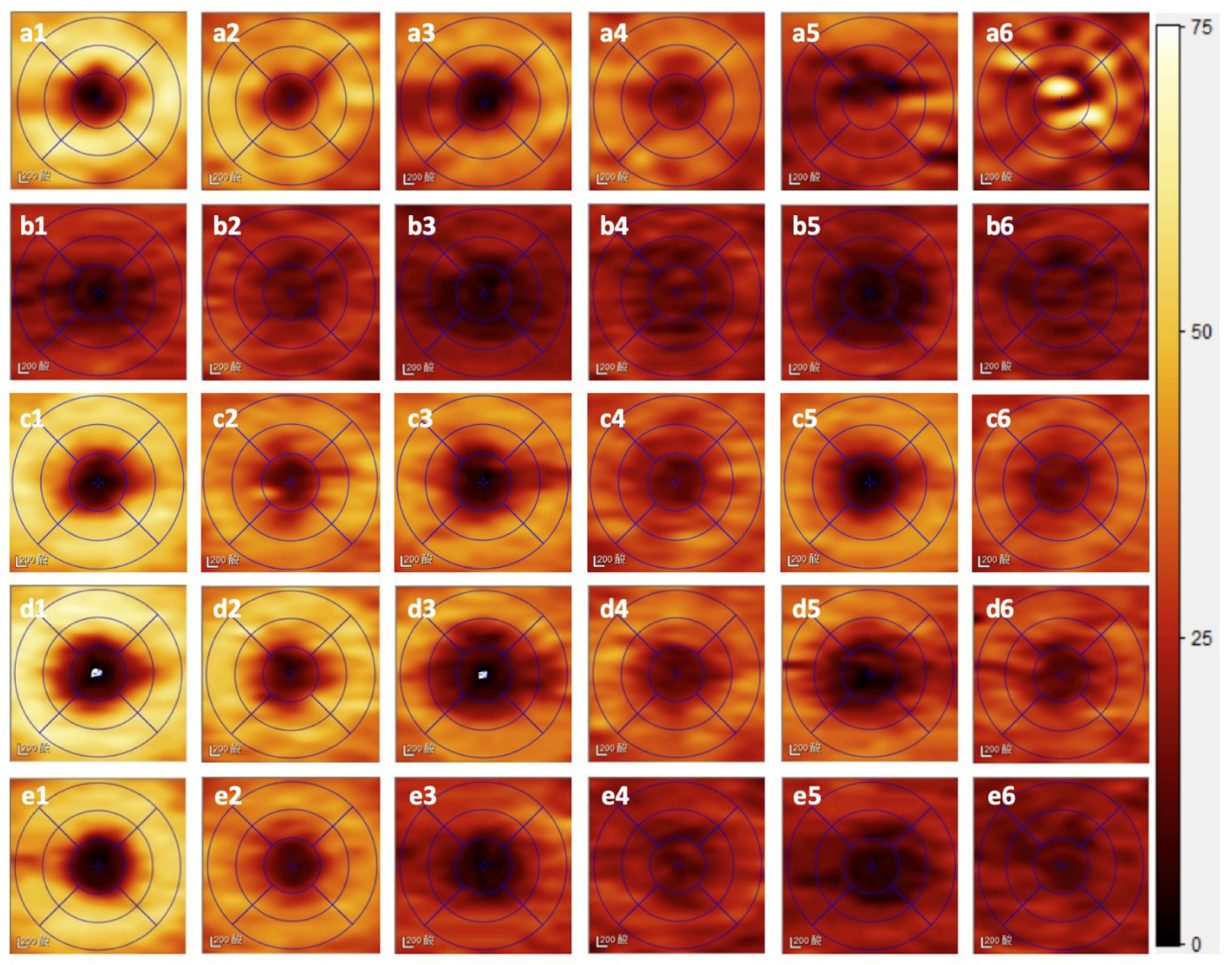
Results of OCT examination. Panels (a–e) indicate the first to the fifth eyes, respectively. Columns 1, 3 and 5 for the mGCL at baseline and 2 and 6 months, respectively. Columns 2, 4 and 6 for mIPL at baseline and 2 and 6 months, respectively. (mGCL = macular ganglion cell layer; mIPL = macular inner plexiform layer).

**Figure 3:**
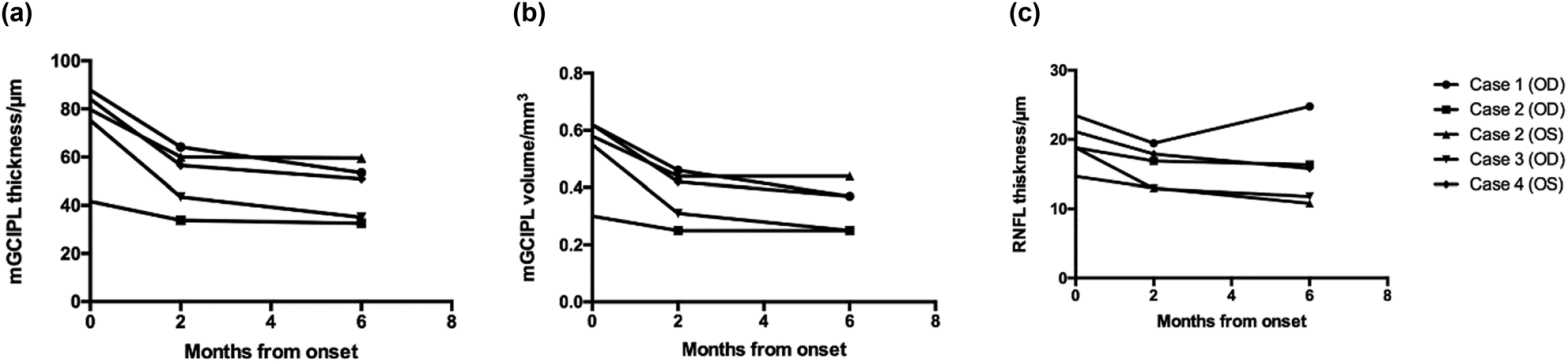
Results of OCT examination: (a) mGCIPL thickness of the affected eye; (b) mGCIPL volume of the affected eye; and (c) RNFL thickness of the affected eye.

**Figure 4:**
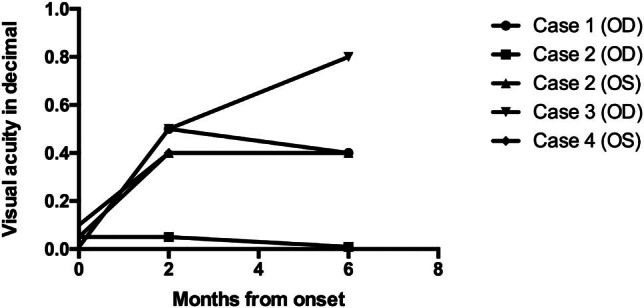
Visual acuity of the affected eye.

We applied the OCT technique to detect the patients’ mGCIPL thickness and volume at the time of onset and 2 and 6 months after onset, which are shown in Figures 2 and 3 and Table 1. The median mGCIPL thickness for the eye of interest at the baseline was 79.67 µm (73.664 ± 18.497 µm, range 41.66–87.77 µm) and the median mGCIPL volume was 0.58 mm^3^ (0.534 ± 0.134 mm^3^, range 0.30–0.62 mm^3^). At 2 months, the median mGCIPL thickness was 60.00 µm (51.576 ± 12.611 µm, range 33.77–64.11 µm) and the median mGCIPL volume was 0.44 mm^3^ (0.376 ± 0.091 mm^3^, range 0.25–0.46 mm^3^). At 6 months, the median mGCIPL thickness was 59.55 µm (46.288 ± 11.876 µm, range 32.55–59.55 µm) and the median mGCIPL volume was 0.44 mm^3^ (0.336 ± 0.084 mm^3^, range 0.25–0.44 mm^3^). In addition, the visual acuity of the affected eyes at the time of onset and 2 and 6 months after onset are illustrated in Figure 4.

### 3.2 Sample size estimation

The sample size was estimated based on the mGCIPL thickness and volume at 2 and 6 months. The detailed results are shown in Table 2.

**Table 2:** Estimation of sample size for mGCIPL volume and thickness

Effect size (%)	80% power				90% power				95% power			
	A		B		A		B		A		B	
	Volume	Thickness	Volume	Thickness	Volume	Thickness	Volume	Thickness	Volume	Thickness	Volume	Thickness
**2 months**												
20	262	256	160	134	350	342	214	180	434	424	264	222
30	118	114	72	60	156	152	96	80	194	188	118	100
40	66	64	40	34	88	86	54	46	110	106	66	56
50	42	42	26	22	56	54	38	30	70	68	44	36
60	30	30	18	16	40	40	24	20	50	48	30	26
**6 months**												
20	140	148	206	166	188	198	274	222	232	244	340	274
30	62	66	92	74	84	88	124	100	104	110	152	122
40	38	38	52	42	52	50	70	56	62	62	86	70
50	24	24	34	28	30	32	44	36	38	40	56	44
60	16	18	24	20	22	22	32	26	26	28	38	32

Effect size: percent decrease in mGCIPL volume and thickness comparing neuroprotective treatment vs GCs arm. A: *Method A*, comparison of follow-up affected eye mGCIPL volume and thickness. The mean and standard deviation values of the mGCIPL volume and thickness of follow-up affected eye were required. Assuming that the protective effects of test drug on mGCIPL volume and thickness were 20, 30, 40, 50 and 60% at 2 and 6 months. The mean values for test drug at 2 and 6 months were calculated according to the different effect sizes of therapeutic effects. Then, the sample size was estimated. B: *Method B*, comparison of difference between follow-up and baseline affected eye mGCIPL volume and thickness. The standard deviation values of this difference were required. The follow-up affected eye in this method was adjusted for baseline affected eye. The other parts were consistent with Method A.

**Figure 5:**
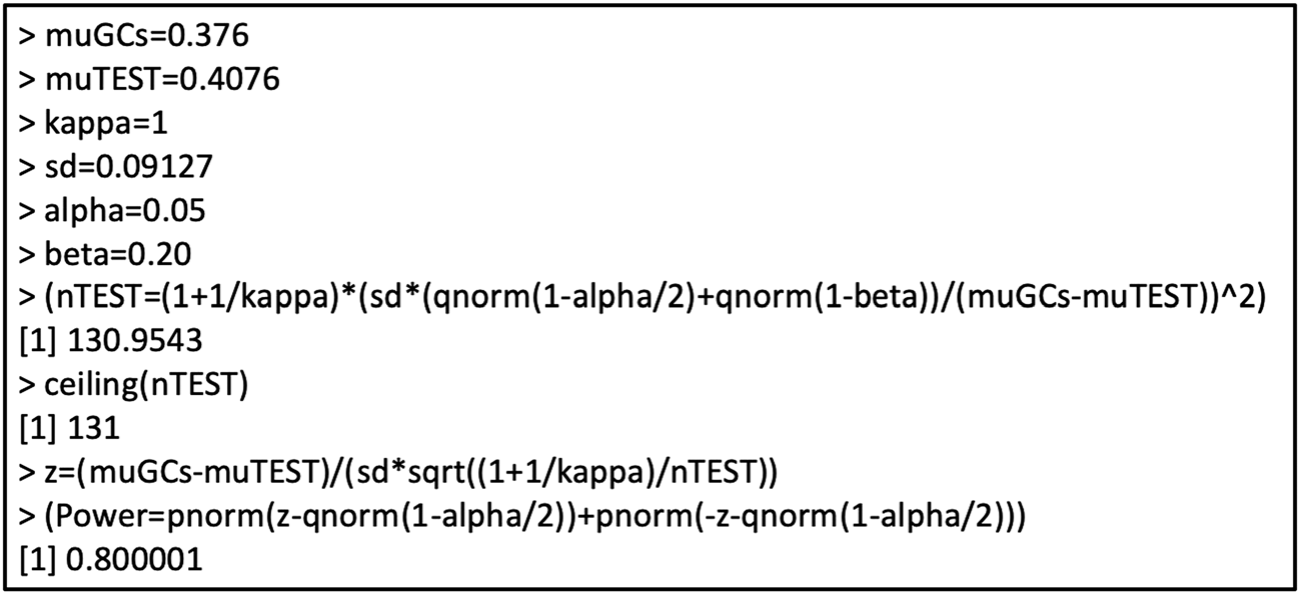
R code for the example of sample size estimate.

As an example:

We aimed to compare a form of neuroprotective drug with GCs regarding the curative effect at 2 months using Method A. Our study showed that in GC treatment, the mean value of the mGCIPL volume at 2 months was 0.376 mm^3^ with an SD of 0.091 mm^3^. We needed to determine the sample size to evaluate the difference between two different groups treated by GCs with placebo and GCs with neuroprotective drug, considering an alpha error of 5% and a beta error of 20%. We assumed that the effect size was 20%, and substituted the values of the means, deviation and alpha and delta errors of the test and the obtained data are shown in Figure 5. Therefore, we needed 262 individuals with AQP4-IgG seropositive ON (131 per arm) to detect the difference between the test drug and GCs. Furthermore, the sample size can be extended according to the estimated loss rate.

## 4 Discussion

Previous research suggested that optic nerve demyelination caused by clinical or subclinical ON results in retrograde degeneration of optic nerve axons, thus leading to RNFL and mGCIPL thinning [11,12]. In this study, we evaluated the changes in the mGCIPL and RNFL from the time of onset to 2 and 6 months after onset of AQP4-IgG seropositive ON patients. As expected, compared with the baseline, both the mGCIPL thickness and volume underwent a clear decline at 2 months after ON onset, whereas no differences were observed between 2 and 6 months, which were similar to the findings reported by Fard et al. [13]. Also, Kupersmith et al. [14] observed mGCIPL thinning at 1 month after ON onset. Even in patients with unilateral disease, the mGCIPL thickness in the contralateral unaffected eyes was gradually reduced during follow-up [15]. However, RNFL thickness measurement could be affected by various conditions, such as varying characteristics of the study subjects and different statistical methods. Fard et al. [13] found that the RNFL thickness underwent a clear decline at 1 month after ON onset. Saidha et al. [16] found a subtype of MS patients with a predominant macular thinning phenotype but normal RNFL thickness. Herein, we found that the RNFL thickness exhibited no obvious difference at the time of onset or 2 or 6 months after ON onset, which may also be explained by the small sample in addition to individual differences. Additionally, it has been found that vascular-mediated optic neuropathy can cause nerve fiber and ganglion cell damage in the inner retina in glaucoma and ischemic optic neuropathy [17]. Kwapong et al. [18] found that decreased macular microvessel density was positively correlated with RGC loss. Therefore, the measurement of mGCIPL thickness and volume is a more specific indicator of RGC loss in ON patients.

The mGCIPL thickness was associated with visual field recovery [7]. Fard et al. [13] also revealed correlations between GCIPL thinning at 1 month and baseline visual acuity, which was in agreement with the association between GCIPL thickness and visual function in MS patients reported by Walter et al. [19]. In the present study, we also assessed the visual acuity of AQP4-IgG seropositive ON patients at the time of onset and at 2 and 6 months after onset. Compared with the baseline, visual acuity underwent clear elevation at 2 months after ON onset, whereas no significant difference was observed between 2 and 6 months, which may be largely due to GC treatment.

The sample size estimation will be helpful for the clinical trials of AQP4-IgG seropositive ON patients. To our knowledge, previous studies have estimated the sample size based on mGCIPL thickness after acute ON [9]; however, AQP4-IgG seropositive and seronegative patients were not distinguished. The etiology and degree of injury in AQP4-IgG seropositive ON patients were different from AQP4-IgG seronegative ON patients, hence AQP4-IgG seropositive patients suffered greater visual impairment, a higher clinical recurrence rate and more clinical demyelinating attacks [20,21]. Additionally, in most previous studies, primary manual analysis of segmented macular layers reduced the repeatability and accuracy of OCT measurements [22]. In the present study, the use of automated mGCIPL volume and thickness measurements was preferred to minimize measurement errors. Although OCT detection alone cannot directly diagnose ON, it can effectively and accurately follow up this disease and may be helpful to predict the prognosis for vision. However, a limitation of this study was the small sample size. Despite this, potential bias could be balanced by the small SD values.

## 5 Conclusion

This study estimated the sample size for two-sample parallel designed clinical trials in AQP4-IgG seropositive ON patients via OCT-measured mGCIPL thickness and volume, which may be helpful for clinical studies in AQP4-IgG seropositive ON patients.
