# Corrigendum to “Sample size estimation for AQP4-IgG seropositive optic neuritis: retinal damage detection by optical coherence tomography”

**DOI:** 10.1515/biol-2025-1335

**Published:** 2026-05-18

**Authors:** Shuwen Lu, Chao Ma, Yi Du

**Affiliations:** Department of Ophthalmology, The First Affiliated Hospital of Henan University of Traditional Chinese Medicine, Zhengzhou, China; Department of Ophthalmology, The First Affiliated Hospital of Zhengzhou University, Zhengzhou, China; Department of Ophthalmology, The First Affiliated Hospital of Guangxi Medical University, Nanning, China

**Original article:**

Lu S, Ma C, Du Y. Sample size estimation for AQP4-IgG seropositive optic neuritis: Retinal damage detection by optical coherence tomography. *Open Life Sciences*. 2024;19:20220866. https://doi.org/10.1515/biol-2022-0866.

**Author approval:**

All authors have reviewed and approved the submission of this corrigendum.

In the published article, Lu S, Ma C, Du Y. “Sample size estimation for AQP4-IgG seropositive optic neuritis: Retinal damage detection by optical coherence tomography.” *Open Life Sciences*. 2024;19:20220866. https://doi.org/10.1515/biol-2022-0866, the authors identified an error in Section 2.2, “AQP4-IgG test and ophthalmologic examinations.”

The sentence:

“The serum was tested for AQP4-IgG by a cell-based assay.”

Should be corrected to:

“Serum aquaporin-4 immunoglobulin G (AQP4-IgG) was tested using a tissue-based indirect immunofluorescence assay on primate cerebellum and optic nerve sections, performed by a commercial laboratory.”

This correction concerns only the description of the AQP4-IgG testing method. It does not affect the optical coherence tomography measurements, sample-size calculations, results, interpretation, or conclusions of the publication.

The authors acknowledge the error and claim that this is an unintentional error that has nothing to do with academic misconduct and does not influence the conclusion of the publication. The authors apologize to the editor, the journal staff, and the readers for the mistake and any inconvenience it caused.

